# Clinical and Sociodemographic Factors Related to Amyotrophic Lateral Sclerosis in Spain: A Pilot Study

**DOI:** 10.3390/jcm13195800

**Published:** 2024-09-28

**Authors:** Belén Proaño, María Cuerda-Ballester, Noelia Daroqui-Pajares, Noemí del Moral-López, Fiorella Seguí-Sala, Laura Martí-Serer, Carlen Khrisley Calisaya Zambrana, María Benlloch, Jose Enrique de la Rubia Ortí

**Affiliations:** 1 Doctoral Degree School, Health Sciences, Catholic University of Valencia San Vicente Mártir, 46001 Valencia, Spain; beprool@mail.ucv.es; 2 Departament of Nursing, Catholic University of Valencia San Vicente Mártir, 46001 Valencia, Spain; maria.cuerda@ucv.es (M.C.-B.); ndelmoral2@mail.ucv.es (N.d.M.-L.); fiorellayada.segui@mail.ucv.es (F.S.-S.); carlenkhrisley.calisaya@mail.ucv.es (C.K.C.Z.); 3 Microbiology Department, General Universitary Hospital of Valencia, 46014 Valencia, Spain; 4 Department of Basic Biomedical Sciences, Catholic University of Valencia, 46001 Valencia, Spain; maria.benlloch@ucv.es (M.B.); joseenrique.delarubi@ucv.es (J.E.d.l.R.O.)

**Keywords:** amyotrophic lateral sclerosis, environmental risk factors, functionality, Spain

## Abstract

**Background:** Amyotrophic lateral sclerosis (ALS) is a neurodegenerative disease of unknow etiology. Male sex is a well stablished risk factor, but other factors such as early and adult life expositions show contradictory evidence. Aim: to explore the link of clinical, sociodemographic, and occupational factors with ALS patients in Spain and the impact of these factors in functionality. **Methods:** A cross-sectional study was conducted with ALS patients and healthy controls. Registered variables were smoking, arterial hypertension, diabetes mellitus type 2, previous cancer to reproductive organs or breast, occupational exposure, and early life exposures. Functionality in ALS patients was compared according to each exposure. **Results:** The ALS group consisted of 59 participants and the control group of 90 participants. ALS patients showed a significant association with previous cancer (*p* = 0.011), occupational exposure (*p* < 0.001), and older siblings (*p* = 0.029). ALS patients presented significant differences in BMI according to hypertension and older-sibling factors. Moreover, respiratory function was affected in patients with previous cancer (*p* = 0.031). **Conclusions:** Occupational exposure and previous cancer to reproductive organs or breast could be linked to ALS patients. In addition, hypertension and previous cancer could affect their BMI and respiratory function. Other factors such as longer smoking periods and exposition to older siblings could also characterize ALS patients.

## 1. Introduction

Amyotrophic lateral sclerosis (ALS) is a progressive neurodegenerative disease that affects motor neurons, leading to a gradual decline in muscle strength and atrophy, ultimately resulting in respiratory failure and death within three to five years post-diagnosis [1]. Currently, ALS is considered a rare disease, with an incidence of 1.68 per 100,000 person-years [2]; Europe has the highest crude incidence among all continents, with significant variations between countries [3]. Specifically, Spain reports a prevalence of 4 per 100,000 inhabitants [4].

The etiology of the majority of ALS cases remains unidentified, but more than 50 genes have been associated with the disease, with missense mutations being the predominant form. Key genes have been identified like SOD1, SETX, TARDBP, FUS, OPTN, UBQLN2, PFN1, TBK1, and NEK1, as well as the hexanucleotide repeat mutation in C9ORF72. In familial ALS cases, which account for approximately 5% of the total cases, these mutations explain 25–35% of the cases, whereas for sporadic ALS, they only represent a contribution of 5–10% [5,6].

Apart from the genetic causes, other intrinsic factors have been proposed, with male sex being well-established as a risk factor [7]. This has raised the possibility of a dysregulation in steroid hormones as an important mechanism for the development of the disease [8,9]. Additionally, another hypothesis suggests that oxidative stress might be a common pathway for neuronal loss in neurodegenerative diseases [10]. 

On the other hand, several lifestyle and environmental factors have been investigated, both in early life [11] and adulthood [12]. Among lifestyle habits, diet and smoking are recognized as significant determinants in the development of various types of cancer [13] and metabolic [14] diseases, which potentially influence thepathological mechanisms underlying neurodegenerative diseases [15,16,17].

Extensive research has been conducted on smoking habits and ALS [18,19,20,21], yet the findings remain inconsistent, with the geographical area being influential. For instance, weak associations have been reported in the United Kingdom [22], no associations in Sweden [23], and a significant association identified through mendelian randomization in a larger European cohort [24]. The mechanisms underlying other medical conditions such as cancer have also produced conflicting results. While cancer overall is not considered a risk factor for ALS, certain types, including melanoma and prostate cancer, may be linked [25,26,27,28]. Other metabolic diseases such as type 2 diabetes mellitus (DM2), hypertension, and hyperlipidemia have garnered some attention, though clear conclusions remain elusive [29,30,31]. Interestingly, a possible protective role of DM2 in relation to ALS has been suggested [32].

Regarding external factors, environmental exposure to neurotoxic compounds (NT) or infectious agents may induce neurotoxicity and neuroinflammation, leading to neuronal death [33]. Such exposure could start in both early life [11] or adulthood, or maybe be related to occupational conditions [34]. These potential risk factors have gained prominence since ALS has been linked to professional physical activity [35] and occupations involving exposure to neurotoxic substances such as pesticides, organic solvents, or heavy metals like selenium [36,37] and lead [38]. Evidence suggest that certain professions, such as health services, veterinarians, teachers, sellers, hairdressers, farmers, and those engaged in strenuous physical work, may constitute a risk factors [34].

The influence of these lifestyle and environmental factors on ALS development may vary among countries due to differences in climate, political context, and dietary practices. Indeed, incidence and prevalence rates differ across European countries [39]. Consequently, it is crucial to gain a deeper understanding of the lifestyle conditions of ALS patients in each region. Research in Europe has focused on countries such as Sweden, Germany, Norway, and Italy, while in Spain—with a different climate and dietary habits—has received comparatively little attention. Thus, the present pilot study aimed to explore the occurrence of the aforementioned clinical and environmental factors in a Spanish sample of ALS patients and to asses whether any of these factors could affect their functionality.

## 2. Materials and Methods

### 2.1. Study Design and Population

An observational, cross-sectional study was conducted with a sample of ALS patients with only bulbar and spinal onsets from different locations of Spain. There was an age-matched control group of people without the disease as a reference for the proportions of the possible risk factors in the population. Both patients and controls were recruited between 2021 and 2022.

The inclusion criteria were as follows: patients with diagnosis of probable or definite ALS according to El Escorial criteria, made at least six months previous to enrollment, who received riluzole treatment; men and women aged over 18 years old. The exclusion criteria were usage of invasive or non-invasive ventilation with positive ventilatory pressure; evidence of dementia; alcohol or drugs dependence; infection with hepatitis B or C; or diagnosis of human immunodeficiency virus. Patients were recruited by random sampling through ALS national associations as part of the clinical trial NCT04654689.

The control group was formed of men and women aged between 40 and 70 years old. Participants were excluded if they presented any neurodegenerative disease. Control participants were recruited by convenience sampling, driven mainly by proximity to hospitals or health centers of the researchers in Spain.

### 2.2. Studied Factors and Functional Evaluation

Information regarding medical history and environmental exposures was collected, together with sociodemographic information, using interview-assisted questionnaires. The sociodemographic variables included were as follows: sex, age, autonomous community of residence, educational level, and primary occupation, including the duration of that occupation. The educational level was recorded as the highest qualification attained: primary, secondary, high school, technical or vocational training, graduate, and post-graduate studies. This was subsequently categorized into three groups: basic (primary and secondary school), middle (technicians—vocational training and baccalaureate), and higher education (university studies—graduate and beyond).

Among the medical history variables, smoking status was reported by selecting from categories of never, former, or current smokers. When applicable, the age at which participants began and quit smoking was also recorded. For analysis, smoking status was simplified into two categories: smoker (including former and current) and non-smoker. Additionally, participants were queried about prior diagnosis of hypertension, DM2, and cancer specific to reproductive organs or breast, due to the potential hormonal alterations suggested for ALS patients [9]. A family history of other neurodegenerative diseases was also documented, focusing on Alzheimer’s disease (AD) and Parkinson’s disease (PD) among parents, grandparents, and siblings.

To assess for environmental exposures during adulthood, the primary occupation was asked as an open-ended question, which was later classified according to the European Skills, Competences, Qualifications and Occupations (ESCO) framework. This classification comprises nine categories of major occupations [40], as outlined in Table 1, with an additional category for housewives. Reported occupations were then classified based on the type of exposure as previously suggested [41]: neurotoxic (NT) compounds, pupils, patients, or customers (Table 1). Occupations were deemed to cause exposure to neurotoxicants if they involved handling pesticides, solvents, or petrol [38], and were classified as causing exposure to infectious agents if they entailed interaction with patients, customers, or pupils [12].

To assess for possible early-life exposures, the age of the parents at the time of the participant’s birth was recorded, with options including under 20 years, between 21 and 30 years, between 31 and 40 years, or older than 40 years. For analysis, parental age was reclassified as either older or younger than 40 years. Finally, the number of siblings and the participant’s birth order were documented, then classified as either last child, with older siblings, or the oldest child with younger siblings, as this could also lead to exposure to infectious agents from younger siblings [11].

Finally, patients underwent a functional assessment using the Revised Amyotrophic Lateral Sclerosis Functional Rating Scale (ALSFRS-R), accompanied by evaluations of respiratory function and body mass index (BMI), as these are stablished prognostic factors of the disease. Forced vital capacity (FVC) and BMI were recorded for each patient.

### 2.3. Statistical Analysis

Statistical analysis was conducted with the program IBM Statistics SPSS v24. Categorical variables were described as absolute (n) and relative (%) frequencies. To test for association of each factor with either control or ALS groups, all variables were recategorized to only two possibilities, and once the sample size was reduced, the Pearson’s chi-squared test or Fisher exact test were used, the latter in case the expected frequency was less than 5 in more than 20% of the cells.

Quantitative variables were described as mean (M) and standard deviation (SD). Normal distribution was assessed with Shapiro–Wilk test. Student’s *t* test, for normal distributed variables, or Mann–Whitney U test, for non-normal distributed variables, were used for functional comparisons between the groups of patients according to each studied factor. This analysis was conducted only when the two groups had more than 5 patients. A linear regression analysis was performed to control for age and sex as confounding factors in the interaction between each functional and predictor variable (only the statistically significant variables that showed differences after the between-groups analysis); to avoid collinearity, the variance inflation factor (VIF) was below 2. Durbin–Watson statistic in each model remained between −2 and 2. The significance level for all tests was α = 0.05.

### 2.4. Ethical Considerations

The data for this study were obtained after approval from the Comité de Ética de la Investigación con Medicamentos (CEIm) from the Hospital Universitario y Politécnico La Fe, Valencia: 2021-001989-38 (9 March 2022) and the Research Ethical Commitee from the Catholic University of Valencia San Vicente Mártir, Valencia: UCV/2021-2022/180 (4 May 2022).

## 3. Results

### 3.1. Comparison between Control Participants and ALS Patients

The ALS group consisted of 59 participants and the control group of 90 participants. The mean age was similar for both groups, at 56 and 55 years, respectively. The autonomous community of residence for the majority of patients was Andalucía at 35.6%; 17% of the patients came from Castilla y León; and 13.6% came from the Community of Madrid and the Valencian Community (Table S1). In the control group, 97% of the participants came from Valencian Community and the rest from Andalucía. 

A comparison of the proportion of men, exposure to NT, each clinical condition, and familiar variables are shown in Table 2. In total, the ALS group comprised 59% men, compared with 51% in the control group. Of the eleven factors studied, only three presented a significant link with the ALS patients: occupational exposure to NT (*p* < 0.001), previous cancer of sexual organs or breasts (*p* = 0.011), and older siblings (*p* = 0.029). On the other hand, smoking, high blood pressure, DM2, parents over 40 years, and a family history of other neurodegenerative diseases presented similar proportions in both groups (*p* > 0.05). Job exposure to other conditions: pupils, customers, and patients is reported in the Supplementary Material (Table S2).

Regarding non-significant factors, smokers made up 64% and 52% in the control and ALS groups, respectively. Both groups had around 20% of patients with high blood pressure, and less than 10% reported DM2. Likewise, the proportion of those with parents over 40 years and relatives with PD was less than 10% for both groups and the proportion of participants with a family history of AD was close to 18%. The proportion of younger siblings almost reached statistical significance, with a trend for a higher proportion in the control group (17%). When the quantitative variables of these factors were considered, no significant differences were found between the groups (Table S3) since both groups reported between 27 and 30 years of work in the reported occupation; participants began smoking at 16 years and developed hypertension after the age of 45; DM2 was not compared since there were only two ALS patients who reported it.

The link between ALS patients and cancer existed in six ALS participants with a previous cancer diagnosis: three men (one with testicular cancer and two with prostate cancer) and three women (two with uterine cancer and one with breast cancer), compared with only one woman with breast cancer in the control group. The link with exposure to older siblings consisted of 17% more patients in this category, compared with the control group. Moreover, the ALS group presented 27% more patients in professions considered at risk for exposure to NT. Table S1 summarizes the reported professions of NT exposure, and the proportion of participants exposed to pupils, customers, and patients.

The comparison of participants working in each ESCO major group is shown in Table 3, where statistical differences were found, with a higher proportion of patients working in categories 2 (professionals), 3 (technicians), and 9 (elementary tasks) and a higher proportion of the control participants in categories 2 (professionals) and 5 (services).

The educational level also was different between the control and ALS groups (Table S1), with 45% of the ALS patients being in the elementary education category, compared with 14% of the subjects in the control group. Additionally, 36% of subject in the control group had a higher education level, compared with 29% of the patients.

### 3.2. Functional Comparison of ALS Patients in Relation to the Factors Studied

**Figure 1:**
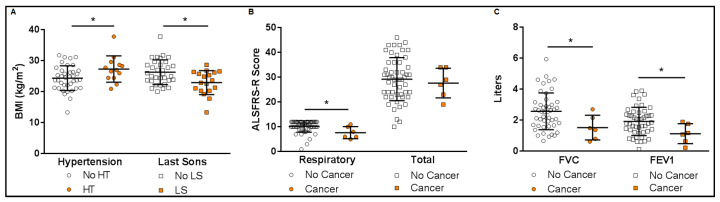
Functional variation in ALS patients in relation to demographic and clinical factors. (**A**). Body mass index (BMI) differences according to hypertension (HT) and last son (LS) conditions. (**B**). ALSFRS-R respiratory and total scores according to previous cancer condition. (**C**). Respiratory capacity according to previous cancer condition. FVC: forced vital capacity, FEV1: forced expiratory volume in 1 s. * *p* < 0.05. Mean and SD bars are presented.

The functionality of ALS patients presented significant differences based on three factors: hypertension, previous cancer of sexual organs or breast, older siblings, and a family history of AD (all comparisons can be seen in the Supplementary Material Table S3). The BMIs of the patients were different depending on whether they had hypertension or not, and whether they had older siblings or not. Thus, hypertensive patients had higher BMIs (*p* = 0.028; Figure 1A) while patients with older siblings had lower BMIs (*p* = 0.005; Figure 1A). On the other hand, a previous cancer diagnosis influenced the respiratory function of the patients, with a lower score in the ALSFRS-R respiratory subscale (*p* = 0.009; Figure 1B) and lower respiratory capacity (Figure 1C): FVC (*p* = 0.031) and FEV1 (*p* = 0.036). Finally, patients who had a mother with AD presented lower scores on the gross motor subscale of the ALSFRS-R (*p* = 0.035; Table S4).

## 4. Discussion

**Figure 2:**
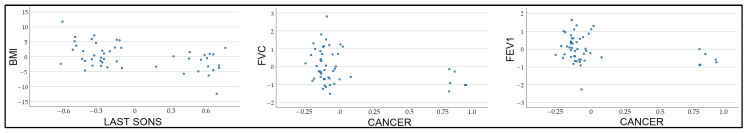
Statistically significant models showing the effect of older siblings in BMI, as well as the effect of previous cancer on respiratory function: FVC and FEV1. Codification of each factor is presented in x axis as zero and one for the absence and presence of the condition, respectively.

The present work aims to analyze the presence of previously suggested risk factors for ALS in a sample of Spanish patients, including a control group of individuals matched by age but without the disease. Finally, when controlling for sex and age, the regression models (Table S5) showed a statistically significant effect of the exposure to older siblings in BMI, even controlling for age and sex. This factor contributed to a 19% variability in the model. In the same way, the previous cancer condition had an effect on respiratory outcomes FVC and FEV1, contributing a 5% and 6% variability to the model in each case, although for these two respiratory variables, sex also had a significant effect, contributing around 40% to the variability of the models (Figure 2).

For the effect of hypertension in BMI, the contribution of the variable remained significant, contributing 10% to the explained variability of the model, although it was not statistically significant in general. Something similar could be seen with the effect of previous cancer in the respiratory subscores of ALSFRS-R.

The job exposure report, as classified in this study, has not been previously presented for Spanish patients. Additionally, the possible implications of these factors on the patients’ functionality and prognostic variables have not been explored before.

The proportion of participants with a history of cancer affecting reproductive organs or the breast was higher in the ALS group compared with the healthy control group. However, these results are inconsistent with previous evidence, which rather suggests that cancer in general is not linked to ALS, and even indicates a trend towards a lower percentage of prostate cancer among ALS patients [28]. These discrepancies may stem from differences in the study designs; the majority of studies assessing mortality risks have employed population-based designs, unlike the present cross-sectional study. Nonetheless, the higher proportion of these specific cancer types could underlie a potential dysregulation in steroid hormones related to testicular cancer [42], breast cancer [43], and ALS [44]. Male ALS patients and those with a specific subset of testicular cancer may exhibit lower testosterone levels compared with controls or other subsets, respectively. For women, the role of steroid hormones in ALS pathology is less clear, although a protective role for estrogen has been suggested [45]. Considering that elevated levels of estrogen and androgen increase the risk of breast cancer, the women with this type of cancer may develop the condition due to causes other than hormonal influences; alternatively, the treatment might have lowered hormone levels to a threshold that increases the risk of developing ALS.

Regarding occupational exposure, there were 27% more people under NT exposure in the ALS group compared with the control group (Table 2). This finding aligns with previous studies that indicate the influence of environmental factors, particularly job exposure, on ALS mortality [40]. Additionally, there was a greater proportion of participants in the ESCO 9 category, which consists of low-skilled jobs characterized by greater physical activity or exposure to toxic compounds. These results are consistent with the work of Pamphlett and Bell [38], in which the majority of patients also belonged to these activity categories: 8 and 9 in the ISCO 2008 classification. The occupations included truck drivers, mechanics, farmers, temporary workers, and those engaged in construction and cleaning activities; these occupations accounted for 22% of the ALS patients in the present sample (Table 3). This supports the potential pathological role of heavy metals and NT compounds found in diesel, organic solvents, and pesticides associated with occupational exposure in these activities [41].

The differential distribution of occupations could also explain the higher prevalence of ALS among men of working age [46], as they predominantly perform many of these risk-related occupations. Previous studies have identified a link between male ALS patients and occupations such as truck driving [38], forestry, and activities related to agriculture, hunting, fishing, and construction [47]. This occupational exposure, combined with specific metabolic and hormonal patterns associated with sex, may predispose men to develop the disease. Furthermore, it is important to note that many studies exclude housewives from occupational databases; however, this activity could be considered a form of exposure to pupils if they are taking care of the children.

Regarding the bias in the region of origin between the control and ALS groups, this may be attributed to the convenience sampling procedure used to recruit the control group. However, it is noteworthy that the most representative communities in the ALS sample correspond to the largest communities according to the latest census from 2021, with Andalucía, Cataluña, the Valencian Community, and the Community of Madrid each having at least five million residents, while Castilla y León had approximately two million [48]. The only exception is Cataluña, which was underrepresented in this study; this is not surprising, considering that this is not a population-based study and patients were recruited for a clinical trial. It has been reported that the sociodemographic characteristics of ALS patients differ between clinical trials and population-based studies [49].

In the present study, Andalucía accounts for more than one third of the sample. Thus, in addition to the population size in each community, it is worth mentioning that Andalucía possess the largest agricultural areas in Spain, where the use of various fungicides, bactericides, and herbicides—primarily based on organic compounds—has been reported [50]. These substances could be considered neurotoxicants, as previously discussed concerning agricultural-related occupations. Although the link between this exposure and ALS has been explored in Andalucia, with no relation detected [50], that study was not a longitudinal study either; therefore, if the job is not considered, exposure is mainly presumed rather than verified, as one individual could reside in a high-exposure area without actual exposure to the neurotoxicant. However, a link between air pollutants and the risk of developing ALS has been reported in the USA [51], Italy [52], and the Netherlands [53].

Additionally, there may be a regional genetic background responsible for the development of ALS in certain areas, as previously suggested for the Catalonian and Valencian regions in Spain [4] and other regions in the world [54].

Among the medical conditions, it was found that hypertension seems to influence the BMI of the patients, with a higher BMI observed in hypertensive patients (Figure 1). However, the proportion of hypertensive patients was not significantly different from that found in the control group (Table 2). This relation is consistent with finding in the general population, where the prevalence of hypertension among individual aged 40 to 59 years increases with rising BMI in both sexes; specifically, a prevalence of 23% and 20% was reported for men and women, respectively, in a BMI range of 25 to 27 kg/m2 [55]. This proportion aligns with the 22% found in patients in the present study, who had an average BMI of 25 kg/m^2^ (Table S4). Thus, deeper studies into the metabolic interactions in the disease could help clarify its pathological processes. Moreover, in the general population, a high BMI is associated with hypertension and elevated levels of total and LDL cholesterol, which in turn increase the risk of cardiovascular diseases [55]. In contrast, for ALS patients, high BMI and LDL values are considered favorable prognostic factors for survival [56].

In this same context of metabolic alterations in ALS patients, previous studies have shown a lower proportion of patients with DM2 compared with the general population, suggesting that DM2 could serve as a protective factor against the disease [29]. Similarly, diagnoses of DM2 and obesity have been associated with a reduced rate of ALS compared with the general population, particularly among men and patients over 60 [57]. In the present study, although the difference in proportions was not statistically significant, there were 9% and 3% of individuals with DM2 in the control and ALS groups, respectively. This could imply that hypertension, typically associated with cardiovascular risk factors such as diabetes, may develop in ALS as a consequence of the disease or in connection with other conditions.

Another relationship identified in our study was the greater proportion of ALS patients exposed to older siblings (Table 2). This finding has not been previously reported and could be due to a socioeconomic condition, wherein the youngest children in large families may have fewer resources and subsequently engage in lower-category work activities. The present sample also exhibited a lower educational level than the healthy controls (Table S2). Conversely, a previous study observed an effect of having younger siblings, suggesting a possible influence of exposure to infectious diseases at an early age that could predispose individuals to develop the disease [11]. This exposure to infections was also explored using the classification suggested by D’Ovidio et al. [12], which categorized exposure to clients (mainly for occupations in the service job categories). In the present study (Table S2), no significant differences were found in the proportions exposed to clients or pupils between the groups, although there were 7% more patients exposed to pupils. In contrast, a higher proportion of controls were exposed to patients, likely due to the sampling source for this group. In any case, the exposure to pathogens from older siblings could also be a possibility; this line of research suggests that viruses, bacteria, or fungi can promote a prion-like protein accumulation in the central nervous system; cause intoxication as observed in Guam cases; or maintain an activated immune response that promotes neuroinflammation [58,59].

Smoking has been a widely investigated risk factor for ALS, showing contradictory evidence [18] that could come from the effect of time spent as smoker, with increasing risk of mortality for smokers of more than 30 years [20]. Furthermore, an effect has been suggested, especially in women [19], again with a reduction of risk occurring when the habit is stopped [20]. It has also been seen that cigarette consumption slightly increases the disease progression rate [21]. In our sample, there were similar proportions of smokers in both groups, around 50–60% (Table 2), but the duration of smoking was slightly higher in ALS patients (*p* = 0.06; Table S3), with almost 30 years, which is consistent with the aforementioned study [20], but there were no statistical differences, possibly because of the reduced sample.

Finally, the combination of functionality and exposure variables (Figure 1) showed a possible influence of previous cancer on respiratory function, with lower FVC and ALSFRS-R respiratory scores observed, even after controlling for age and sex. This finding is particularly significant, given that respiratory failure is the main cause of death [60], and should be considered when assessing the disease progression. Furthermore, a family history of mothers with AD was also associated with lower gross motor skills (Table S4). To our knowledge, these results have not been previously described and may represent part of a common neurodegeneration pattern. It also could be a statistical finding by chance. It is also noteworthy that smoking did not appear to effect FVC functionality, contrary to what was expected. 

It is crucial to pursue in-depth studies on environmental exposures and lifestyle habits, as they are modifiable factors that could be mitigated by reducing exposure to neurotoxicants, especially in Spain, which has a high agricultural production in Europe. In general, other job-related exposures could also be minimized through improved conditions for basic occupational categories. Additionally, gender differences in the division of labor should be considered as part of the interplay with physiological variations that could trigger the development of the disease in men and women.

## 5. Limitations of the Study

The present study provides valuable information about ALS and the intrinsic and environmental factors that may be present in a Spanish sample; however, it has certain limitations. As a cross-sectional study, the use of analytical measurements such as ORs lacks validity. The small sample size also precluded an analysis of the interaction of factors or the use of designs that control for confounding variables, as there would be too many categories without participants. In addition, small sample sizes increase the probability of type II errors. Beyond statistical limitations, the control and ALS groups were not sufficiently homogenous, exhibiting differences in educational level that could weaken the conclusions related to job exposure. This may reflect a bias in the sampling, where only individuals with higher educational levels and better jobs participated in the survey. Considering these limitations, it is strongly recommended to conduct a longitudinal study with a larger sample size and improved quantification of the level and duration of exposure to the potential risks, as well as a more precise classification of the region of origin, differentiating between rural or urban areas.

## 6. Conclusions

The medical and environmental conditions reported in this Spanish sample are consistent with previous findings regarding job exposure to NT compounds, particularly in specific occupations such as transportation, hairdressing, agriculture, and cleaning activities. ALS patients could also exhibit a longer duration of smoking than healthy controls. Furthermore, hypertension and a history of cancer in reproductive organs or the breast could impact the BMI and respiratory capacity of ALS patients. Finally, there may be a potential influence of older siblings and a family history of mothers with AD on ALS predisposition and disease progression, which warrant further investigation.

## Figures and Tables

**Table 1:** Classification of occupations based on ESCO classification.

Code	Major Group *	Exposure	
0	Forced armed occupations	NT:	Gas station workers (ESCO: 3, 4, 5) Drivers and mechanics (ESCO: 8, 9) Cleaning activities (ESCO: 8, 9) Hairdressing and hairdressing vocational teachers (ESCO: 23, 51) Farmers and forestry activities (ESCO: 6, 9) Construction, chemical and paint production activities (ESCO: 7) Military service (ESCO: 0)
1	Managers		
2	Professionals		
3	Technicians and associate professionals		
4	Clerical support workers		
5	Service and sales workers		
6	Skilled agricultural, forestry and fishery workers		
7	Craft and related trades workers		
8	Plant and machine operators and assemblers	Pupils:	Teachers of primary, secondary, and higher-level education (ESCO: 23) Housewives
9	Elementary occupations	Patients:	Health professionals and assistants (ESCO: 22, 32, 532)
10	Housewives	Customers:	Service and selling activities (ESCO: 32, 34, 35, 41, 42, 52)

* Adapted from [40], NT: neurotoxic compounds.

**Table 2:** Age and proportion of exposed subjects in control and ALS groups.

	Control	ALS	Test	*p*-Value
	(M ± SD)	(M ± SD)		
Age	55 ± 8	56 ± 10	u	0.428
	n (%)	n (%)	test	*p*-value
Men	46 (51)	35 (59.3)	chi	0.260
Smokers	59 (64)	31 (52.5)	chi	0.157
Hypertension	17 (18.5)	13 (22)	chi	0.593
DM2	8 (8.7)	2 (3.4)	f	0.317
Cancer	1 (1.1)	6 (11)	f	0.011
Exposure NT	11 (12.2)	23 (39)	chi	<0.001
Younger siblings	40 (52)	20 (35)	chi	0.052
Older siblings	14 (18.4)	20 (35)	chi	0.029
Mother +40	2 (2.2)	4 (6.8)	f	0.212
Father +40	7 (7.6)	5 (8.5)	f	>0.999
PD relatives	4 (4.3)	4 (6.8)	f	0.712
AD relatives	16 (17.4)	11 (18.6)	chi	0.845

M: mean, SD: standard deviation, NT: job exposure to neurotoxic compounds, DM2: diabetes mellitus type 2, +40: older than 40, PD: Parkinson disease, AD: Alzheimer disease; u: Mann–Whitney U test, chi: chi-squared, f: Fisher exact test.

**Table 3:** Occupations of the control and ALS participants according to ESCO.

ESCO	Control (*n* = 90) *n* (%)	ALS (*n* = 59) *n* (%)	*p*-Value *
0	1 (1)	1 (2)	0.006
1	0	3 (5)	
2	26 (29)	14 (24)	
3	19 (21)	11 (19)	
4	1 (1)	0	
5	30 (33)	8 (14)	
6	0	2 (3)	
7	1 (1)	4 (7)	
8	3 (3)	3 (5)	
9	6 (7)	10 (17)	
10	3 (3)	3 (5)	

ESCO: major group classification; * *p*-value from Fisher exact test.

## Data Availability

Data are contained within the article.

## Supplementary Materials

The following supporting information can be downloaded at: https://www.mdpi.com/article/10.3390/jcm13195800/s1, Table S1. Autonomous community of procedence of the ALS patients in the study sample; Table S2: Educational level and job exposition in control and ALS groups; Table S3: Time of exposure to the studied factors; Table S4: Functional comparisons between patients with or without each one of the studied factors; Table S5. Regression models for each functional variable controlling for age and sex.
