# Supplementary material for: Clinical and Sociodemographic Factors Related to Amyotrophic Lateral Sclerosis in Spain: A Pilot Study
Source: J Clin Med. 2024 Sep 28;13(19):5800. doi: 10.3390/jcm13195800 (PMC11476538; doi:10.3390/jcm13195800)
Supplement: Supplementary file 1 [file jcm-13-05800-s001.zip › jcm-3141209-supplementary.pdf]

Table S1. Autonomous community of procedence of the ALS patients in the study sample.

Autonomous community	n (%)
Andalucía	21 (35.6)
Aragón	2 (3.4)
Islas Balerares	1 (1.7)
Canarias	2 (3.4)
Castilla y León	10 (17)
Castilla-La Mancha	3 (4.3)
Cataluña	1 (1.7)
Comunidad Valenciana	8 (13.6)
Extremadura	1 (1.7)
Galicia	1 (1.7)
Comunidad de Madrid	8 (13.6)
País Vasco	1 (1.7)
Total	59

Table S2. Educational level and job exposition in control and ALS groups.

Education level	Control (n = 90)	ALS (n = 59)	***p***
Basic	13 (14 %)	25 (42 %)	< .001*
Middle	45 (50 %)	17 (29 %)	
Higher	32 (36 %)	17 (29 %)	
Job exposed	Control (n =61)	ALS (n = 43)	
NT	11 (12.2 %)	23 (39 %)	< 0.001
Pupils	7 (8 %)	9 (15 %)	0.149
Patients	30 (33 %)	4 (7 %)	< 0.001
Customers	12 (13 %)	7 (12 %)	0.793

*p-value from Chi-squared test: $X^2{}_{2,\,0{,}05} = 15{,}23$.

Table S3. Time of exposure to the studied factors.

	Control<br>M ± SD	ALS<br>M ± SD	test	p-value
Occupation (y)	26.9 ± 10.8	29.9 ± 13.5	u	0.148
Smoking (a)	17.1 ± 2.7	16.3 ± 2.3	u	0.170
Smoking (y)	23.5 ± 13.4	28.9 ± 12.5	t	0.065
Hypertension (a)	50.5 ± 8.2	46.2 ± 8.3	u	0.208
DM2 (a)	47.3 ± 7.9	53.5 ± 2.1	-	-

M: mean, SD: standard deviation, y: years; a: age of beginning or diagnosis, t: student's t test, u: Mann-Whitney U.

Table S4. Functional comparisons between patients with or without each one of the studied factors.

	No		Yes		
Job exposure	**M**	**SD**	**M**	**SD**	**p**
ALSFRS-R bulbar	9.9	2.3	9	3.8	0.656
ALSFRS-R fine	4.6	3.8	5.3	3.9	0.487
ALSFRS-R gross	4.7	3.3	4.7	3.6	0.888
ALSFRS-R resp	10	2.5	9.8	2.3	0.697
ALSFRS-R total	29.2	7.7	29	9.7	0.902
BMI	25.8	4.2	24	4.2	0.121
FVC	2.4	1.1	2.5	1.4	0.797
Smoking					
ALSFRS-R bulbar	9.7	3	9.5	3.1	0.788
ALSFRS-R fine	4.3	3.8	5.4	3.8	0.21
ALSFRS-R gross	4.5	3.4	4.9	3.5	0.619
ALSFRS-R resp	10	2.2	9.9	2.7	0.969
ALSFRS-R total	28.4	7.9	30	9	0.574
BMI	25.7	4.4	25	4.1	0.392
FVC	2.3	1.2	2.6	1.2	0.499
Hypertension					
ALSFRS-R bulbar	9.3	3.2	11	1.8	0.118
ALSFRS-R fine	4.9	3.8	4.8	4.2	0.832
ALSFRS-R gross	4.7	3.6	4.5	2.8	0.897
ALSFRS-R resp	10.2	2.3	8.9	2.8	0.127
ALSFRS-R total	29.1	8.7	29	7.8	0.938
BMI	24.4	4	27	4.3	0.028
FVC	2.5	1.2	2.3	1.1	0.702
Cancer					
ALSFRS-R bulbar	9.7	3.1	9.2	3.1	0.534
ALSFRS-R fine	5.1	3.8	6.2	3.8	0.597
ALSFRS-R gross	4.9	3.4	4.7	4.2	0.807
ALSFRS-R resp	10.3	2.3	7.7	2.4	**0.009**
ALSFRS-R total	29.9	8.7	28	6	0.541
BMI	24.8	3.7	25	4.9	0.864
FVC	2.6	1.2	1.5	0.8	**0.031**

	No		Yes		
Younger siblings	**M**	**SD**	**M**	**SD**	**p**
ALSFRS-R bulbar	9.2	3.2	10.4	2.3	0.131
ALSFRS-R fine	4.7	3.7	5.6	4.1	0.523
ALSFRS-R gross	4.8	3.5	4.7	3.5	0.96
ALSFRS-R resp	9.8	2.5	10	2.4	0.441
ALSFRS-R total	28.5	8.7	30.7	8	0.351
BMI	24.5	4.7	26.3	3.2	0.182
FVC	2.4	1.2	2.6	1.2	0.284
Older siblings					
ALSFRS-R bulbar	9.9	2.9	9.2	3.2	0.338
ALSFRS-R fine	5.1	3.9	4.9	3.7	0.84
ALSFRS-R gross	4.4	3.5	5.5	3.4	0.219
ALSFRS-R resp	9.8	2.6	10	2.1	0.632
ALSFRS-R total	29.2	8.7	29.4	8.3	0.93
BMI	26.5	4	23	3.9	**0.005**
FVC	2.3	1.1	2.8	1.4	0.257
AD relatives					
ALSFRS-R bulbar	9.9	2.6	8.3	4.3	0.361
ALSFRS-R fine	5	3.9	4.6	3.7	0.852
ALSFRS-R gross	5.1	3.4	3	3.3	**0.035**
ALSFRS-R resp	9.9	2.6	10.1	1.5	0.618
ALSFRS-R total	29.8	8.5	26	7.9	0.181
BMI	25.3	4.3	24.5	4.1	0.618
FVC	2.5	1.2	2.1	1	0.326

Table S5. Regression models for each functional variable controlling for age and sex.

**Outcome: BMI**			
Predictors	b	β	p-value
Age	-0.05	-0.11	0.460
Sex	-1.50	-0.17	0.209
Hypertension	3.10	0.32	0.027
Model: F = 2.43; R^2 = 0.13; ΔR^2 = 0.10; p = .07			
Predictors	b	β	p-value
Age	-0.07	-0.17	0.219
Sex	-2.19	-0.25	0.060
Last sons	-4.13	-0.47	**0.001**
Model: F = 4.79; R^2 = 0.24; ΔR^2 = 0.19; p = .006			

**Outcome: ALSFRS-R respiratory subscore**			
Predictors	b	β	p-value
Age	0.00	0.00	0.996
Sex	-0.35	-0.07	0.583
Cancer	-2.59	-0.34	0.015
Model: F = 2.34; R^2 = 0.12; ΔR^2= 0.11; p = 0.084			

**Outcome: FVC**			
Predictors	b	β	p-value
Age	-0.01	-0.08	0.478
Sex	-1.44	-0.60	**<.001**
Cancer	-0.94	-0.26	**0.027**
Model: F = 12.42; R^2 = 0.45; ΔR^2 = .06; p <.001			

**Outcome: FEV1**			
Predictors	b	β	p-value
Age	-0.01	-0.12	0.303
Sex	-1.07	-0.58	**<.001**
Cancer	-0.67	-0.23	**0.044**
Model: F = 11.48; R^2 = 0.42; ΔR^2 = 0.05; p <.001			

Beta (b) and beta standardized (β) coefficients are presented for each factor, as well as the F, R^2 and p-value for each model. Statistically significant effects are in bold.
